# Hip resurfacing arthroplasty: A systematic review of functional outcome

**DOI:** 10.3109/17453674.2010.501742

**Published:** 2010-11-26

**Authors:** Marijke van Gerwen, Daniel A Shaerf, Remmelt M Veen

**Affiliations:** ^1^Department of Orthopaedic Surgery, Rijnland Hospital, Leiderdorp, the Netherlands; ^2^Department of Plastic and Reconstructive Surgery, Royal Free Hospital, London, UK; ^3^Department of Orthopaedic Surgery, Sint Antonius Hospital, Nieuwegein, the Netherlands

## Abstract

**Background and purpose:**

Hip resurfacing arthroplasty is claimed to allow higher activity levels and to give better quality of life than total hip arthroplasty. In this literature review, we assessed the therapeutic value of hip resurfacing arthroplasty as measured by functional outcome.

**Methods:**

An extensive literature search was performed using the PubMed, Embase, and Cochrane databases.

**Results:**

9 patient series, 1 case-control study, and 1 randomized controlled trial (RCT) were included. Clinically and statistically significant improvement in sporting activity and hip scores were found in 10 studies.

**Interpretation:**

Studies with low levels of evidence have shown improvement in various different hip scores and one RCT showed better outcomes with hip resurfacing arthroplasty. There is no high-level evidence to prove that there is improved clinical outcome using hip resurfacing arthroplasty. More randomized research needs to be done.

During the mid-1990s, new designs of hip resurfacing arthroplasties (HRAs) were introduced. These implants use a metal-on-metal bearing surface with the advantage that there is no polyethylene wear debris. The medium- to long-term results of this new generation of prosthesis seem promising, with few complications (Treacy et al. 2005, Buergi and Walter 2007). Although these metal-on-metal bearings produce less wear debris, the long-term consequences of metal release are unknown. This is especially important since HRA is being used in younger individuals. Studies have shown an increase in plasma levels of chromium and cobalt of about 2–13 times for chromium and 4–7 times for cobalt (Trentani and Vaccarino 1981, Vendittoli et al. 2007). Furthermore, the formation of pseudotumors as a consequence of metal-on-metal prostheses has been reported (Mahendra et al. 2009, von Schewelov and Sanzén 2010).

It is claimed that patients can return to their previous activity level and even perform high-impact sports (Infozimmerdurom 2008, Smith & Nephew 2008, DePuy 2008). We therefore investigated the evidence for this claim. We asked: “What is the therapeutic value of HRA in patients who have undergone HRA for osteoarthritis, measured by quality of life or functional outcomes?”

## Methods

In December 2008, we performed a literature search using the PubMed and Embase databases. The search was performed only on the basis of determinant, because of a limited amount of literature on the subject; different synonyms for resurfacing HRA were used (Table 1, see Supplementary data). Search queries were limited to Title/Abstract. Relevant studies were screened for references. Articles in languages other then English, Dutch, French, or German and articles with other domains, determinants, or outcomes were excluded. Further studies were selected according to relevance and were critically appraised according to criteria developed by the Center for Evidence-Based Medicine, Therapy Worksheet, n.d.

## Results

**Figure 2.**
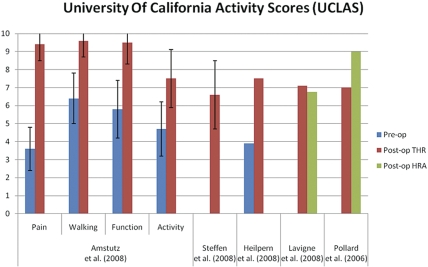


**Figure 3.**
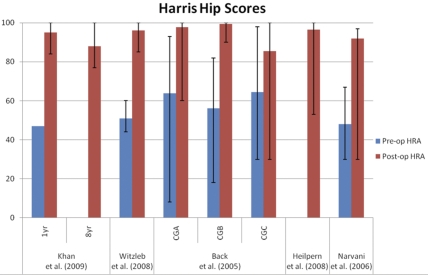


The search using the PubMed, Embase and Cochrane databases yielded 245 articles (Figure 1, Supplementary data). Screening on Title/Abstract resulted in 23 articles, and after exclusion of duplicates 15 articles remained. No additional articles were found by cross-referencing. After selection by relevance and critical appraisal, 11 articles remained for inclusion. Articles were considered relevant if they included the domain “patients with osteoarthritis”, determinant “HRA” and outcome “clinical outcome” and were aimed at answering our research question. 9 studies were patient series, 1 was a case-control study, and 1 was a randomized controlled trial, which was ranked highest. 3 patient series contained over 500 patients and 2 studies contained more than 200 patients. Median or mean follow-up was less than one year for 2 studies, 1–5 years for 5 studies (which included the randomized controlled trial), and > 5 years for 4 studies. All patient series showed good clinical outcome scores (Table 3). Pollard et al. (2006) found significantly better outcome scores for Birmingham hip replacement (BHR) than for total hip arthroplasty (THA). Lavigne et al. (2008) found better outcome scores for HRA than for THA, of which only the differences in global activity scores were statistically significant. Tables 2 and 3 and Figures 2 and 3 give an overview of the results.

**Table 2.** General study data

Author	Follow-up	Mean age	Women (%)	Men (%)	Complication	Revision
Khan et al. (2009)	mean 6 (5–8) years	median 51 (16–88)	40	60	4%	29 (4.4%)
Amstutz et al. (2008)	mean 5.6 (1.1–11) years	50 (14–78)	25.3	74.4	8.1%	34 (3.4%)
Heilpern et al. (2008)	mean 71 (60–93) months	54 (35–75)	42	58	4%	4 (3.5%)
Lavigne et al. (2008)	minimum 1 year	48 (23–63)	35/28 **^b^**	65/72 **^b^**	unknown	unknown
Steffen et al. (2008)	mean 4.2 (2.0–7.6) years	52 (17–82)	41	59	3.8%	23 (3.8%)
Witzleb et al. (2008)	median 24 (2–66) months	49 (15–69)	43	57	3.4%	2 (0.8%)
Naal et al. (2007)	mean 23.5 (9–40) months	53	–	–	unknown	unknown
Narvani et al. (2006)	minimum 6 months	unknown	–	–	unknown	unknown
Pollard et al. (2006)	mean 61 (52–71) months	50 (18–67)	25/22 **^b^**	75/78 **^b^**	11(21%)/7(11%) **^b^**	1 (1.9%)/4 (6.3%) **^b^**
Back et al. (2005)	median 36 (25–52) months	52 (18–82)	35	65	10% **^a^** (5.6%)	1 (0.43%)
McMinn et al. (1996)	mean 8.3 (1–19) months	unknown	–	–	unknown	0

**^a^** superficial wound infections included.

**^b^** THA/HRA

**Table 3.** Results of clinical scores

Author	Preoperative			Follow-up		
	UCLA score	HHS	Other scores	UCLA score	HHS	Other scores
Khan et al. (2009)	47				95 (84–100) 1-yr 88 (77–100) 8-yr	DAS 6 (5–6) 95% extremely, satisfied at 7 years
Amstutz et al. (2008)	3.6 (SD 1.2) pain; 6.4 (SD 1.4) walking; 5.8 (SD 1.6) function; 4.7 (SD 1.5) activity			9.4 (SD 0.9) pain; 9.6 (SD 0.9) walking; 9.5 (SD 1.2) function; 7.5 (SD 1.6) activity		
Heilpern et al. (2008)	3.9 (1–10)		OHS 41.9 (16–57)	7.5 (4–10)	96.4 (53–100)	OHS 15.4 (12–49)
Lavigne et al. (2008)			GAS HRA 5.8 GAS THA 5.1	HRA 7.1 THA 6.75		GAS HRA 17.9 GAS THA 12.4 WOMAC HRA 8.1 WOMAC THA 9.8
Steffen et al. (2008)				6.6 (SD 1.9)		OHS 16.1 (SD 7.7)
Witzleb et al. (2008)		51 (44–60)			96 (85–100)	
Naal et al. (2007)			4.8 (SD 2.3) different sports			4.6 (SD 1.9) sports (NS) 85% excellent/good
Narvani et al. (2006)		48 (30–67)	65% sports participation		92 (30–97)	92% sports participation
Pollard et al. (2006)				THA 7 (3–10) HRA 9 (4–10)		Sports: THA 33.3% HRA 73.6% EQ-VAS: THA 69.3 HRA 82.3
Back et al. (2005)		CA 63.9 (8–93); CB 56.2 (18–82); CC 64.4 (30–98)	SF12 31.1; 58.6 SF12 30.3; 60.5 SF12 31.5; 52.2		97.7 (60–100) 99.4 (90–100) 85.5 (30–100)	SF12 54.1; 56.9 SF12 54.1; 57.7 SF12 48.2; 55.9
McMinn et al. (1996)			Charnley scores: Pain 3 Mobility 3.1 Walking 3.3			Pain 5.4 Mobility 5.4 Walking 5.4

## Discussion

In every study, patients and clinicians knew which prosthesis was used; therefore, all studies were subject to certain degrees of bias, most of all confounder bias since validated outcome scores were not used. The study of Pollard et al. (2006) was a case-control study but they restricted patients who received THA in their activities. This also introduces bias. The studies by Amstutz and Le Duff (2008) and Khan et al. (2009) received funding from industry-related companies and institutions. As with any new complex surgical technique, there is a learning curve associated with the surgeon's technical ability to perform the procedure. A publication bias may therefore exist, as only surgeons at the top of their learning curve, operating regularly, would obtain sufficient numbers to warrant publication of their cases. Finally, our review suffers from the same publication bias that all literature reviews are subject to: negative findings are less likely to be published.

The studies included quoted variable complication rates ranging from 3% to 10%. This 3-fold difference can be partly explained by the different definitions used by the authors regarding what constituted a complication. Only 4 of the studies had a follow-up period of longer than 5 years. Revision rates were also reported by Khan et al. (2009) as 4%, by Amstutz and Le Duff (2008) as 3%, by Heilpern et al. (2008) as 4%, by Steffen et al. (2008) as 4%, by Witzleb et al. (2008) as 0.8%, by Pollard et al. (2006) as 2% for THA and 6% for HRA, by Back et al. (2005) as 0.4%, and by McMinn et al. (1996) as 0% (Table 4). Although not part of our study aim, these numbers suggest relatively high revision rates with short follow-up. In a study performed by Hallan et al. (2007) on uncemented THA with a minimum follow-up time of 7 years and an endpoint of revision of aseptic loosening of the stem, the survival was between 96% and 100% at 10 years. So complication and revision rates in the studies included are already relatively high. We can only assume that complication rates would be higher with longer follow-up. The literature on THA suggests that augmented revision rates in this type of surgery only start to rise after 7 years.

The use of different clinical outcome scores and the differences in the duration of follow-up make direct comparison between studies difficult. A combination of the University of California Los Angeles activity scale (UCLAS) and Harris hip score (HHS) was used in 5 studies as follow-up scores, but UCLAS was only used in 1 study preoperatively and HHS in 4 (Table 3). We found only 2 studies that directly compared HRA with THA. Reviewing the available literature, there is evidence that HRA has a better outcome than THA (as measured by HHS or UCLAS). Beaulé et al. (2006) performed a study on 152 patients who underwent THA with an average age of 59 (21–87) years, and an average follow-up of 5 (2–21) years. They found a mean HHS of 89 (SD 14) (range: 40–100) and mean UCLAS score was 6.8 (2–10). Similar outcome scores were found by Moran et al. (2004) (with a mean HHS of 85 (SD 14) for consultants and of 85 (SD 14) for trainees) and by Lieberman et al. (1997) (with a mean HHS of 85 (26–100)).

### Conclusion

Our review shows that HRA does have a good clinical outcome with respect to function in the short to medium term, based on data from the 11 available studies. However, the level of this evidence is generally poor with most studies (9) being level 4. Pollard et al. (2006) restricted the activity of the control (THA) group, leading to a distinct possibility of bias. Only 1 study was a randomized controlled trial (Level 1c) involving 209 hips. The functional outcome measures of HHS and UCLAS appear to convey a clinically significant improvement compared to those found in the literature for THA, but there have been very few studies that performed a direct comparison between the two treatment modalities. The clinical outcomes of revision rates and complications are surprisingly high in some of the studies identified here, with relatively short follow-up. Long-term results from these studies are eagerly awaited, to see whether the benefits identified withstand the test of time as robustly as those of THA without a disproportionately high revision rate over a similar follow-up period.

## Supplementary data

Supplementary Figure 1 and Table 1 are available at our website (www.actaorthop.org), identification number 3590/10.
