# Supplementary material for: Hip resurfacing arthroplasty: A systematic review of functional outcome
Source: Acta Orthop. 2010 Nov 26;81(6):680–3. doi: 10.3109/17453674.2010.501742 (PMC3216077; doi:10.3109/17453674.2010.501742)
Supplement: Supplementary file 1 [file ORT-1745-3674-81-680-s3590.pdf]

Supplementary article data

Hip resurfacing arthroplasty

A systematic review of functional outcome

Marijke van Gerwen[1], Daniel A Shaerf[2], and Remmelt M Veen[3]

[1]Department of Orthopaedic Surgery, Rijnland Hospital, Leiderdorp, the Netherlands, [2]Department of Plastic and Reconstructive Surgery, Royal Free Hospital, London, UK, [3]Department of Orthopaedic Surgery, Sint Antonius Hospital, Nieuwegein, the Netherlands
Correspondence: ma.vangerwen@gmail.com
Submitted 009-07-26. Accepted 10-03-06

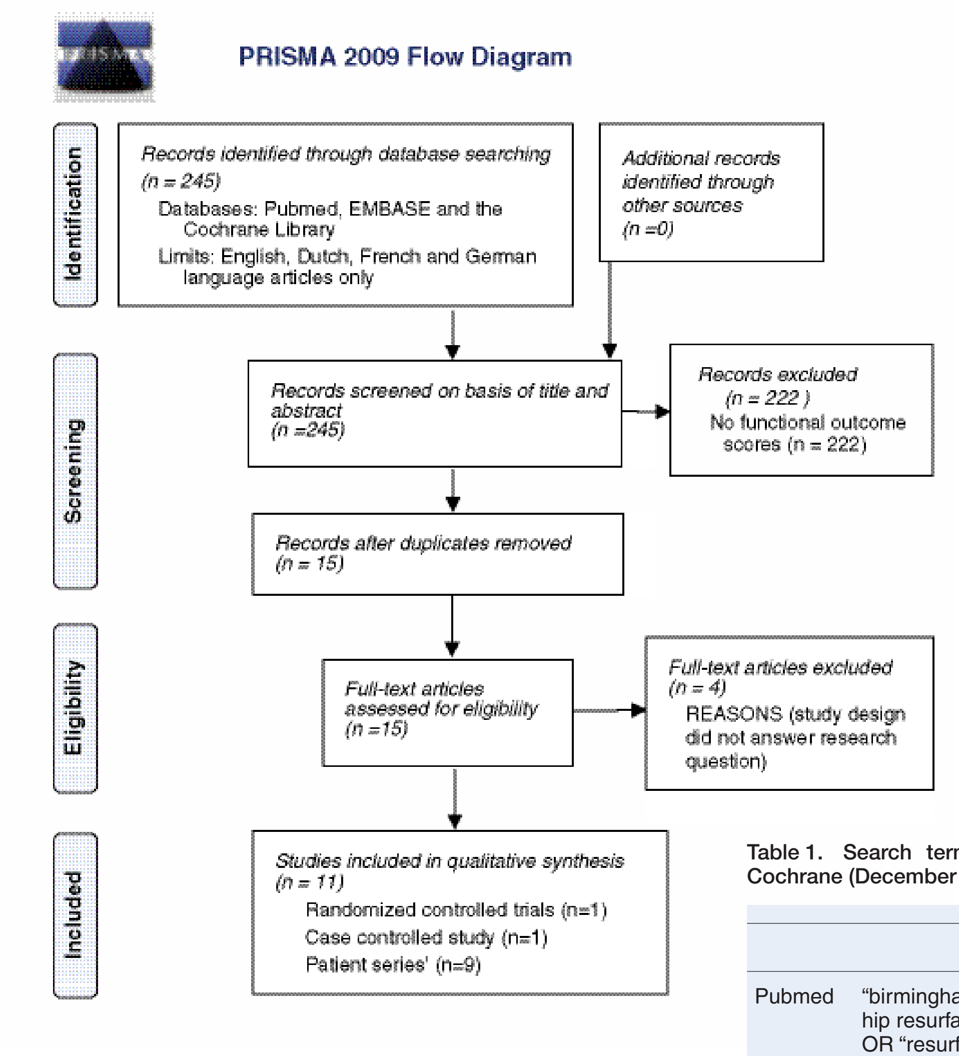

Table 1. Search terms and results for Pubmed, Embase and Cochrane (December 2008)

	SEARCH	RESULTS
Pubmed	"birmingham hip resurfacing*" OR "birmingham hip resurfacings"OR "resurfacing hip arthroplasty" OR "resurfacing hip prostheses" OR "resurfacing hip replacement" OR "resurfacing implant" OR "femur revision prosthesis" OR ("resurfaced" AND "hip") OR "articular surface replacement"	132
Embase	"	111
Cochrane	"resurfacing hip arthroplasty"	2
TOTAL		245

Open Access - This article is distributed under the terms of the Creative Commons Attribution Noncommercial License which permits any noncommercial use, distribution, and reproduction in any medium, provided the source is credited.
DOI 10.3109/17453674.2010.501742
